# Comparison of Transcriptome Differences in Soybean Response to Soybean Mosaic Virus under Normal Light and in the Shade

**DOI:** 10.3390/v11090793

**Published:** 2019-08-29

**Authors:** Lei Zhang, Jing Shang, Wenming Wang, Junbo Du, Kai Li, Xiaoling Wu, Liang Yu, Chunyan Liu, Muhammad Ibrahim Khaskheli, Wenyu Yang

**Affiliations:** 1 Sichuan Engineering Research Center for Crop Strip Intercropping System, College of Agronomy and Key Laboratory for Major Crop Diseases, Sichuan Agricultural University, Chengdu 611130, China; 2 National Center for Soybean Improvement, National Key Laboratory for Crop Genetics and Germplasm Enhancement, Key Laboratory of Biology and Genetic Improvement of Soybean, Ministry of Agriculture, Nanjing Agricultural University, Weigang 1, Nanjing 210095, China; 3 Department of Plant Protection, Sindh Agriculture University, Tandojam 70060, Pakistan

**Keywords:** soybean, soybean mosaic virus, shading, RNA-Seq, plant-pathogen interaction

## Abstract

Shading in the intercropping system is a major abiotic factor which influences soybean growth and development, while soybean mosaic virus (SMV) is a biotic factor that limits the yield and quality of soybean. However, little is known about the defense response of soybean to SMV in the shade. Thus, in the current study, both intensity and quality (red:far-red, R:FR) of the light were changed to simulate the shaded environment and comparative transcriptome analysis was performed. Morphologically, plant growth was inhibited by SMV, which decreased 35.93% of plant height and 8.97% of stem diameter in the shade. A total of 3548 and 4319 differentially expressed genes (DEGs) were identified in soybean plants infected with SMV under normal light and in the shade. Enrichment analysis showed that the plant defense-related genes were upregulated under normal light but downregulated in the shade. Pathways that were repressed include plant-pathogen interaction, secondary metabolism, sugar metabolism, and vitamin metabolism. In addition, genes associated with signaling pathways such as salicylic acid (SA), jasmonic acid (JA), and ethylene (ETH) were also downregulated in the shade. A qRT-PCR assay of 15 DEGs was performed to confirm transcriptome results. According to our knowledge, this is the first report on soybean response to dual stress factors. These results provide insights into the molecular mechanisms in which soybean plants were infected with SMV in the shade.

## 1. Introduction

Due to the advantages of interspecific complementation, nutrient exchange, marginal effects, and high biodiversity, maize-soybean strip intercropping has become the main cultivation model for soybean in southwestern China [1,2,3]. However, during the symbiosis of maize and soybean, the light environment was changed, as well as disease resistance of soybean [3,4].

Soybean mosaic virus (SMV) is one of the major pathogen causing severe yield loss and is widely distributed worldwide [5]. SMV is a member of the *Potyvirus*. The genome of SMV is approximately 10 kb in length and encodes 11 mature proteins [6,7]. These proteins work together to successfully attack the plants. After infection with the virus, the leaves of the plant produce symptoms such as mosaic, shrinkage, and deformity. This may be related to damage of chloroplast structure in the host plant by virus infection, which further causes the chlorosis of leaves [8]. The dwarfing is another significant phenotypic change in soybean after SMV infection. Studies have shown that the viral infections cause changes in host photosynthesis, hormone transduction, and cell wall function [9,10,11,12,13]. By studying the soybean V1 leaves (the first trifoliate leaf) after infection with SMV at 7, 14, and 21 days post-inoculation (dpi), it was found that the defense response to the virus was activated at later stage, which helped the virus to establish a systemic infection [14]. This is consistent with the study by Zhao et al. [15]. In another study, Díaz-Cruz et al. performed a transcriptome analysis of soybean V2 leaf infected with SMV at 10 dpi and found that differentially expressed genes (DEGs) were predominantly upregulated. The host defense responses and signal transduction were significantly induced, while energy metabolism and photosynthesis were repressed [16].

Close planting and intercropping alter the light environment, including light intensity (Photosynthetically active radiation, PAR) and light quality (red:far red-light ratio, R:FR) in the field [17]. Light changes are sensed by photoreceptors. Among them, phytochrome B (phyB) plays a major role in sensing R:FR [18]. In the shade, phyB is regulated by a reduced R:FR, causing shade intolerant plants to activate the transcription of growth-promoting genes [19]. In response, these plants accelerate the elongation of hypocotyls and petiole, erect leaves, reduce branching, and premature flowering, a phenomenon known as the shade avoidance syndrome (SAS) [20]. As a result, plant defenses are reduced because limited resources are allocated for growth [21]. Many studies have shown that plants display weak defense against pathogen infection in shade conditions or FR-enriched conditions. *Arabidopsis* in the shade conditions represses jasmonate-dependent defense by regulating the protein stability of MYC transcription factor (TF) and its jasmonate ZIM-domain (JAZ) repressors [22]. Low R:FR reduces the resistance of *Arabidopsis* to *Botrytis cinerea* [23]. Light deficiency increases the susceptibility of *Nicotiana benthamiana* to turnip mosaic virus (TuMV) infection [24]. In addition, after the phytochrome are mutated, plant resistance is reduced even under normal light conditions. The accumulation of pathogenesis-related 1 (PR1) protein in the rice phytochrome triple mutant (*phyAphyBphyC*) was significantly attenuated, and the plants were more sensitive to blast fungus [25]. Compared to wild-type plants, phytochrome-deficient *Nicotiana tabacum* has reduced resistance to cucumber mosaic virus (CMV) [26] and chilli veinal mottle virus (ChiVMV) [27].

Under the maize-soybean intercropping system, the light intensity and light quality (especially for R:FR) received by soybean canopy were severely reduced due to the shade of high maize [17]. In our previous survey, the incidence of viral diseases in intercropped soybean was much lower than that of monoculture crop, but the severity of the disease was increased. In the case of reduced light intensity and light quality, the defense response of soybean to SMV, especially at the transcriptional level, has not been studied yet. In the present study, we used high-throughput RNA-Seq to compare DEGs in SMV-inoculated soybean plants under normal and shaded light conditions. Under very high shade conditions, the sensitivity of soybean to virus increased. To the best of our knowledge, this is the first transcriptome study on light-regulated soybean response to virus infection.

## 2. Materials and Methods

### 2.1. Plant Material, Virus Inoculation, and Light Treatment

Soybean seeds (Nannong 1138-2, a SMV susceptible variety) were kindly provided by Dr. Kai Li from Nanjing Agricultural University in China. The SMV isolate (YA87) was collected from the field soybean plants in Sichuan Province, China. The bean *Phaseolus vulgaris* cv. Topcrop was used for local-lesion purification of SMV, and then the virus was propagated on the soybean cv. Nannong 1138-2 [28]. Soybean seeds were surface-sterilized and sown in a mixed matrix containing PINDSTRUP organic soil (Pindstrup Mosebrug A/S, Ryomgaard, Denmark) and vermiculite (*v:v*, 4:1) in an artificial climate chamber with 25 °C/22 °C day/night temperature, 60% relative humidity and 14 h/10 h of photoperiod. The light condition for seedling growth was PAR of 296.92 µmol m^−2^ s^−1^ and R:FR of 5.92. The two unifoliolate leaves of 10-day-old soybean plants were mechanically inoculated with virus inoculum, which was homogenized by SMV-infected leaves in 0.01 mol L^−1^ K-phosphate buffer (pH 7.4). The mock treatments were inoculated with phosphate buffer without SMV. After inoculation, a part of the plants was transferred to a shaded environment and the other part was kept under normal light. The shading condition was PAR of 50.14 µmol m^−2^ s^−1^ and R:FR of 0.55, which was simulated by covering the green filter (type No. 122, Q-MAX, England) and adding far-red light-emitting diode (36 W, light peaking at 735 nm). The following four treatments were applied: Control under normal light (NC), SMV infection under normal light (NS), control in the shade (LC), and SMV infection in the shade (LS).

### 2.2. Sample Collection and Illumina Sequencing

Previous studies have shown that larger changes in transcriptional levels occurred in soybeans infected SMV at 10 dpi [16]. Therefore, we collected the V2 leaves (the second trifoliolate leaf, newly grown) of soybean plants after treatment for 10 dpi. Total RNA was extracted using phenol-chloroform-isoamyl alcohol and lithium chloride, washed using 70% ethanol, and finally checked by Agilent 2100 Bioanalyzer to ensure RIN number > 7.0. After the samples were tested, cDNA libraries were constructed and paired-end sequencing was performed based on the Illumina HiSeq 2500 platform at the Personal Biotechnology Co., Ltd. (Shanghai, China). Three biological replicates were set up for each treatment and a total of 12 independent samples were used for RNA-Seq.

### 2.3. Read Alignment and Expression Analysis

The reads number, Q30, N (%), Q20 (%), and Q30 (%) of raw data were counted. After removing reads containing sequencing adapters and reads of low quality, the clean data were mapped to the reference genome of *Glycine max* (Glyma2.0) using Bowtie2 and Tophat2. The reads mapped to exon region were also counted. HTSeq (Version 0.11) was used to calculate the read count mapped to each gene as the initial expression level of the gene. Gene expression levels were normalized using the RPKM (reads per kb per million reads) method. Differential expression analysis between treatments was identified by DESeq2 with screening parameters of log_2_FC (fold change) > 1 and *p*-adj (adjusted *p*-value) < 0.05.

### 2.4. Functional Enrichment Analysis of DEGs

The latest genomic reference information of *Glycine max* was obtained from the Soybase (www.soybase.org), including Gene Ontology (GO) annotations for each gene. The Kyoto Encyclopedia of Genes and Genomes (KEGG) annotations were obtained from the KEGG database. A hypergeometric test was used to find out the GO terms and KEGG pathways that were significantly enriched by DEGs. The enrichment analyses of GO and KEGG were performed using the OmicShare online website (www.omicshare.com/tools).

### 2.5. Validation of Gene Expression by qRT-PCR

To verify the accuracy and reproducibility of the RNA-Seq data, qRT-PCR assays were conducted with gene specific primers. Total RNA from the same treated samples were extracted. Reverse transcription was performed using 5× All-In-One RT Master Mix kit (AccuRT Genomic DNA Removal Kit, ABM, Vancouver, Canada). In addition, 2× ChamQ Universal SYBR qPCR Master Mix (Vazyme, Nanjing, China) was used and Eppendorf Mastercycler ep realplex (Eppendorf, Hamburg, Germany) instrument was used for the qRT-PCR experiment. Each treatment contained three independent biological replicates and three technical replicates. The expression level of soybean *β*-actin gene was used as an internal reference. The fold change value of gene expression was calculated using the 2^−ΔΔCt^ method. The sequences of specific primers were listed in Table S1.

## 3. Results and Discussions

### 3.1. Plant Phenotypes and Virus Detection after Inoculation

**Figure 1:**
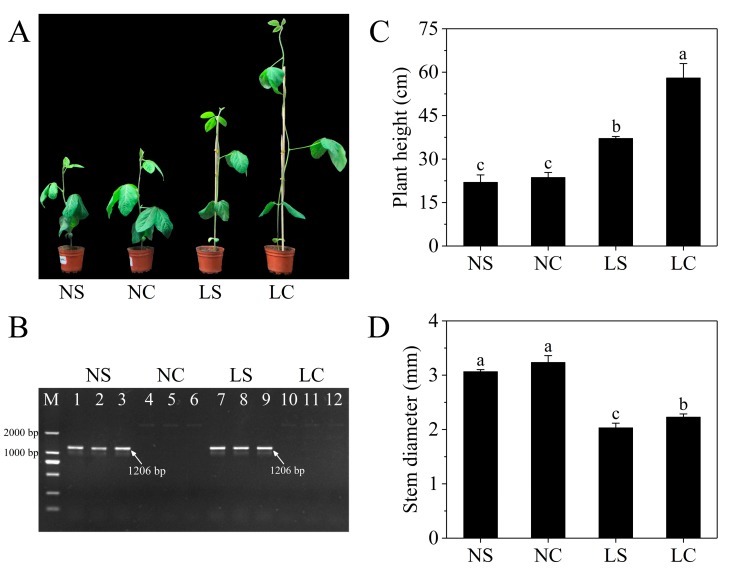
Plant phenotypes and soybean mosaic viris (SMV) detection after inoculation. (**A**) Soybean seedlings grown for 10 days after infection with SMV under different treatments. (**B**) RT-PCR detection to determine SMV infection. M: DNA Marker; 1–3: SMV infection under normal light (NS) plants; 4–6: Normal light (NC) plants; 7–9: SMV infection in the shade (LS) plants; 10–12: Control in the shade (LC) plants, respectively. (**C**) Plant height after 10 days of SMV infection; (**D**) Stem diameter after 10 days of SMV infection. Three independent experimental replicates were analyzed for each treatment, and data are indicated as the mean ± SE. The means for each treatment that do not have a common letter are significantly different at *p* = 0.05, according to Duncan’s multiple range test. NS and NC refer to SMV infection and control, respectively, under normal light, while LS and LC refer to SMV infection and control, respectively, in the shade.

Soybean seedlings of 10 days were mechanically inoculated with SMV and subsequently grown under normal light (PAR, 296.92 µmol m^−2^ s^−1^ and R:FR, 5.92) and in the shade light (PAR, 50.14 µmol m^−2^ s^−1^ and R:FR, 0.55), respectively. After 10 days of treatment, the growth of the plants was affected by the viruses and light conditions, and the leaves showed typical mosaic symptoms. The plant height and petiole length of the plants were significantly elongated in the shade (Figure 1A). However, after SMV infection, the development of the plant was impaired, and both the plant height and stem diameter were reduced (Figure 1C,D). RT-PCR demonstrated that the virus-inoculated plants were SMV-positive (Figure 1B).

Plant growth and defense are often negatively correlated because growth and development are affected by the infection of the pathogen [29]. The phenomenon of viral infection leading to dwarfing of plants is often observed [16,30,31]. In our experiments, soybean seedling growth and development was impaired after infection with SMV. In the shade, plant growth is more severely damaged, with a reduction of 35.93% of plant height and 8.97% of stem diameter. Similarly, Díaz-Cruz et al. studied soybean infected with SMV for 10 dpi and found that the average plant height was reduced to 26% as compared to the control (CK) [16]. This may be an important factor in the reduction of yield in the shade, that is, the growth and development of plants are seriously affected by the virus.

### 3.2. Evaluation of RNA-Seq Data

To investigate the changes in the levels of gene expression in response to SMV infection under normal light and in the shade, we performed transcriptome sequencing of soybean plants. A total of 12 independent cDNA libraries were generated, which included triplicates of four treatments: SMV infection (NS) and control (NC) under normal light, SMV infection (LS), and control (LC) in the shade. The raw data are shown in Table S2. The Q20 percentages of raw data were above 95.62%. Both clean reads and clean base percentages were greater than 98% (Table S3). Overall, the reads total mapped on the reference genome were more than 83%, and the uniquely mapped reads were over 93% (Table S4). It should be noted that the mapping percentage of LS2 was lower compared to other samples, which might be affected by sample preparation or sequencing. Statistics on mapping to genomic regions showed that the proportion of mapping to exon regions accounted for more than 97%, including LS2 (Table S4). These data indicated that reliable transcriptome data was available for subsequent differential analyses.

### 3.3. Statistics on the Number of DEGs

**Figure 2:**
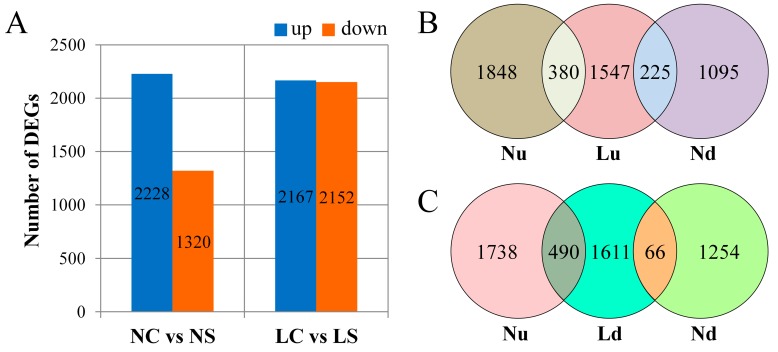
Differentially expressed genes (DEGs). (**A**) Number of DEGs between SMV infection and CK. (**B**,**C**) Venn diagrams showing the number of overlaps in DEGs. NS and NC refer to SMV infection and control, respectively, under normal light, while LS and LC refer to SMV infection and control, respectively, in the shade. Nu and Nd refer to upregulated genes and downregulated genes between NC vs NS. Lu and Ld refer to upregulated and downregulated genes between LC vs LS.

DEGs were screened out between the treatments with log_2_FC (fold change) > 1 and *p*-adj (adjusted *p*-value) < 0.05. Under the normal light, a total of 3548 DEGs were identified between soybean leaves infected with SMV and CK, of which 2228 were upregulated and 1320 were downregulated. Under shading treatment, a total of 4319 genes were affected, including 2167 upregulated and 2152 downregulated (Figure 2A). This suggested that a large number of genes were downregulated when the soybean was infected with SMV in the shade. In order to clearly observe the effect of light on DEGs, we performed overlapping analysis on genes that were upregulated and downregulated, respectively. Overall, 380 genes (Nu-Lu, Figure 2B) were upregulated and 66 genes (Nd-Ld, Figure 2C) were downregulated under two light treatments. However, 225 genes were downregulated under normal light but upregulated in the shaded light (Nd-Lu, Figure 2B). In contrast, 490 genes were upregulated under normal light but downregulated in the shade light (Nu-Ld, Figure 2C). This indicated that the normally activated genes of the plant were suppressed when the light was insufficient.

### 3.4. GO Function Enrichment Analysis of DEGs

**Figure 3:**
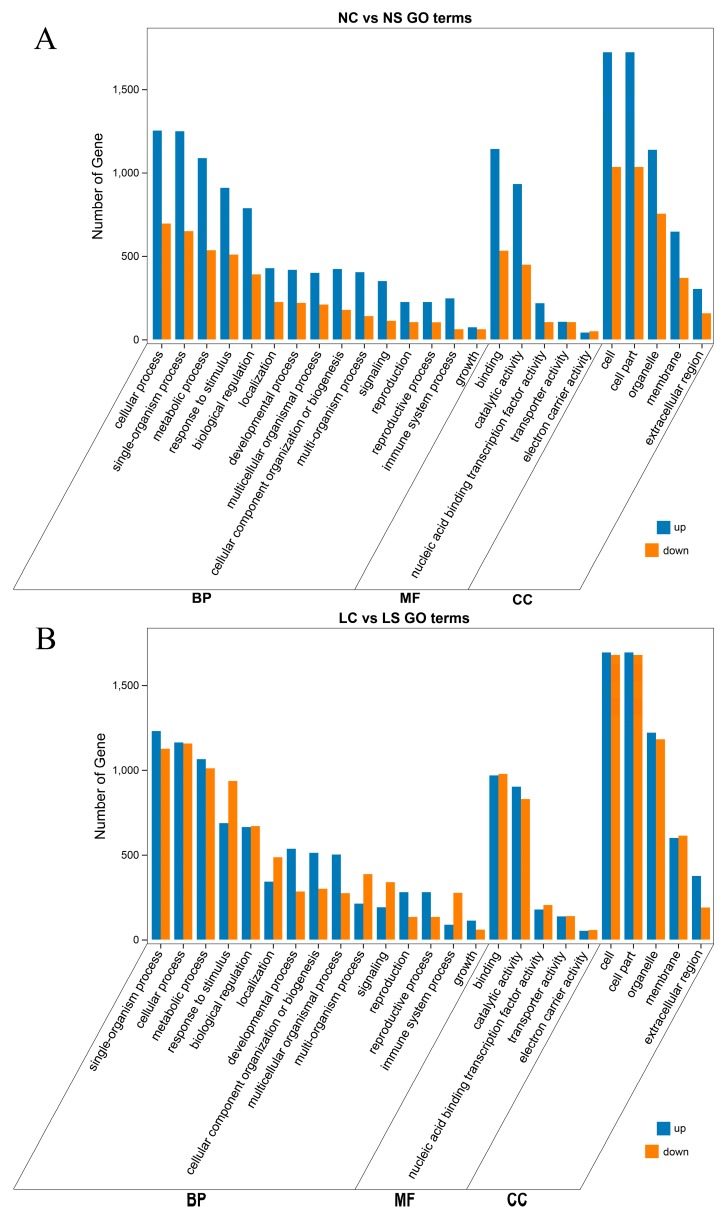
Gene Ontology (GO) Function Enrichment Analysis of DEGs identified. (**A**) GO analysis of DEGs between NC vs NS. (**B**) GO analysis of DEGs between LC vs LS. NS and NC refer to SMV infection and control, respectively, under normal light, while LS and LC refer to SMV infection and control, respectively, in the shade. MF, BP, and CC refer to molecular function, biological process, and cellular component.

Gene Ontology (GO) enrichment analysis was used to determine the functional classification of DEGs between different treatments. Genes were divided into three categories: Biological process, molecular function, and cellular component. Among them, the enrichment terms of the biological process were the most common. The functional classification of DEGs after viral infection was very similar, in both normal light (Figure 3A) and shade (Figure 3B). For example, the cellular process, single-organism process, metabolic process, response to stimulus, and biological regulation in biological process, the binding and catalytic activity in molecular function, and cell and cell part in cellular component. The difference was observed in the shade, where the number of genes that were downregulated was greater (Figure 3B). Obviously, in response to stimulus, signaling and immune system process, DEGs were mainly upregulated under normal light. However, in the shade, the numbers of downregulated genes were increased dramatically, even more than the number of upregulated genes. This suggested that light had a positive regulatory effect on the expression of immune-related genes.

### 3.5. KEGG Pathway Enrichment Analysis of DEGs

**Figure 4:**
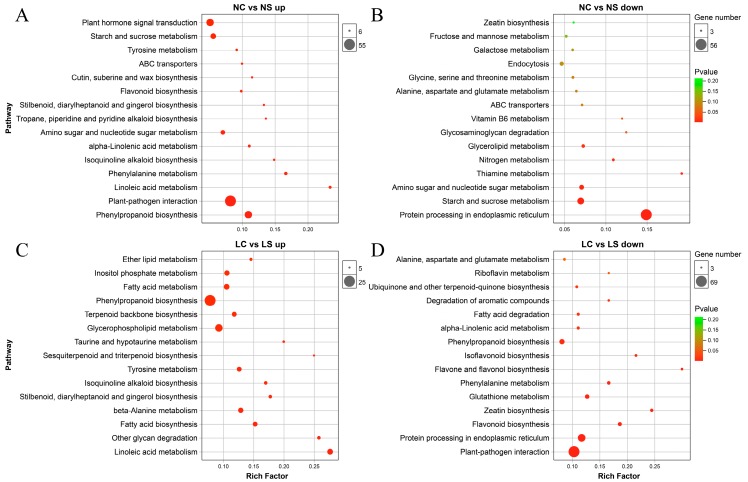
Kyoto Encyclopedia of Genes and Genomes (KEGG) Pathway enrichment analysis of DEGs identified. KEGG Pathway analysis based on (**A**) the differentially upregulated genes and (**B**) downregulated genes between NC vs NS. KEGG Pathway analysis based on (**C**) the differentially upregulated genes and (**D**) downregulated genes between LC vs LS. NS and NC refer to SMV infection and control, respectively, under normal light, while LS and LC refer to SMV infection and control, respectively, in the shade.

Kyoto Encyclopedia of Genes and Genomes (KEGG) enrichment analysis of DEGs was performed to determine changes in metabolic pathways following the viral infection and light treatment. Figure 4 showed the top 15 enrichment pathways for each group of DEGs. Under normal light, the most significant phenylpropanoid biosynthesis was induced, followed by plant-pathogen interaction, linolenic acid metabolism, and phenylalanine metabolism, while the protein processing in endoplasmic reticulum was repressed, as well as sugar metabolism (starch and sucrose metabolism, glycosaminoglycan degradation, galactose metabolism, and fructose and mannose metabolism), nitrogen metabolism, and vitamin metabolism (thiamine metabolism and vitamin B6 metabolism) (Figure 4A,B). In the shade, Linoleic acid metabolism, other glycan degradation, and fatty acid biosynthesis were induced. However, the plant-pathogen interaction, protein processing in endoplasmic reticulum, and secondary metabolism (flavonoid biosynthesis, zeatin biosynthesis, isoflavonoid biosynthesis, and flavone and flavonol biosynthesis) were repressed (Figure 4C,D). Like GO enrichment, plant immune-related pathways of soybean plants were activated in normal light but inhibited in shading conditions, indicating the positive regulation of light on plant defense mechanism.

It was described that the genes related to metabolism, proteins with binding function, development, and defense were differentially expressed when the soybean was infected with SMV [14]. Also, in previous studies, it was shown that at 10 days after infection with SMV, defense response, and signal transduction related genes in soybean were significantly induced, while energy and photosynthesis genes were repressed [16]. These studies are consistent with the results of our study. Phenylalanine is a precursor of a series of secondary metabolites. It plays an important role in response to stress. Defensive pathways including plant-pathogen interaction were greatly activated under the normal light. However, in the shade, the plant-pathogen interaction was significantly inhibited, along with secondary metabolism and vitamin metabolism. The secondary metabolites, such as flavonoid and isoflavonoid, have been documented to be important in stressed resistance [32,33]. Vitamins, some small molecular compounds, play an important role in the integrity of biological functions. Previous studies have shown that after sweet potato was infected by virus, the vitamin biosynthesis process was repressed [34].

### 3.6. DEGs Involved in Plant-Pathogen Interaction

A total of 76 DEGs were enriched to plant-pathogen interaction pathway under normal light conditions, which includes 55 upregulated and 21 downregulated genes (Figure S1 and Table S5). Under the shading treatment, 83 genes were differentially expressed, of which 15 were upregulated and 69 were downregulated (Figure S2 and Table S6). Among these, 24 genes were differentially expressed under both light conditions, including WRKY transcription factor (GLYMA_18G056600, GLYMA_18G208800, GLYMA_03G042700), MYB transcription factor (GLYMA_05G234600, GLYMA_09G038900, GLYMA_19G214900, GLYMA_20G209700, GLYMA_10G180800, GLYMA_02G244600), Enhanced Disease Susceptibility 1 (GLYMA_06G187300, GLYMA_06G187400), and calcium-binding protein (GLYMA_06G034700, GLYMA_14G222000, GLYMA_02G059600, GLYMA_14G156300). Most of these overlapped genes were induced under normal light but repressed in the shade (Table 1).

The genes involved in plant immunity and pathogen infection constitute the plant-pathogen interaction pathways [35]. Effective regulation of plant defense systems is the basis for successful resistance to pathogens. WRKY and MYB are transcription factors involved in plant stress resistance under both biological and abiotic stresses [36,37,38]. An R2R3-MYB transcription factor has effect on tomato yellow leaf curl virus infection in tomato [39]. CaM is a Ca^2+^-binding protein that plays a role in developmental and stress responses. When tobacco was infected with TMV, NtCaM1, NtCaM2, and NtCaM13 were accumulated before induction of PR1 and PR3 expression [40]. EDS1, indispensable in the SA defense path, has been reported [41,42]. This series of regulatory genes was downregulated when plants were infected with SMV in the shade, suggesting that the plant’s defense network cannot be activated in the shade.

### 3.7. DEGs Involved in Plant Hormone Signal Transduction

Plant hormones play an important role in plant growth and development. Some of these hormones are essential for plant immunity [43]. It is well-known that salicylic acid (SA), jasmonic acid (JA), and ethylene (ETH) play a leading role in disease resistance. We analyzed the DEGs of these three hormone pathways in the current study. Under normal light, the plants activated genes from three pathways after 10 days of SMV infection as show in Figure S3. The induction of ethylene-responsive transcription factor ERF (GLYMA_10G186800) and PR1 (GLYMA_15G062300) were the largest among others (Table 2). However, DEGs were mainly suppressed in the shade conditions (Figure S4). The most downregulated ERF (GLYMA_02G006200) had a log_2_FC value of −6.71, followed by PR1, with a Log_2_FC value of −5.91. The MYC and JAR of the JA pathway were also significantly suppressed. These results indicated that the defense hormone regulatory network was inhibited when soybean plants was infected with SMV in the shade.

SA is an important hormone involved in the regulation of resistance to biotrophic and hemibiotrophic pathogens such as plant viruses [44]. In the current study, the SA pathway was repressed when soybean plants were infected with SMV in the shade conditions. It has been studied that light is an important factor for the regulation of plant defense signaling pathways [21]. Both light intensity and light quality can affect the expression of the PR gene in plant-pathogen interaction systems [24]. Phytochromes positively regulated *Nicotiana tabacum* against CMV [26] and ChiVMV [27] via a salicylic acid-dependent pathway. In addition, changes in the light conditions can also affect the JA pathway, which modulates the plant resistance to pathogens, such as *Podosphaera xanthii* [45], *Pseudomonas syringae* [46], *Magnaporthe grisea* [25], and *Botrytis cinerea* [23]. In this study, JA signal pathway of soybean plants was repressed in the shade. Previous studies have reported that ETH may act as a regulator of SA and JA pathway [47]. There are few reports on the effects of light on the ETH pathway. In this study, many ERF genes were found downregulated, suggesting that the ETH may also have an important role in regulating the defense in shade.

### 3.8. Validation of RNA-Seq Data by qRT-PCR

To further confirm the gene expression pattern obtained from RNA-Seq, 15 DEGs were selected for qRT-PCR, including Phytochrome kinase substrate 1 (GLYMA_01G046600), A-ARR (GLYMA_04G247800), BAK1 (GLYMA_05G119500), AUX1 (GLYMA_12G030900), PIF4 (GLYMA_14G032200), PR1 (GLYMA_15G062400, GLYMA_15G062500, and GLYMA_15G062700), PhyB (GLYMA_15G140000), JAR1 (GLYMA_16G026900), HSP70 (GLYMA_17G072400), WRKY62 (GLYMA_18G056600), PhyA (GLYMA_20G090000), DELLA (GLYMA_20G200500), and ERF1 (GLYMA_20G203700).

**Figure 5:**
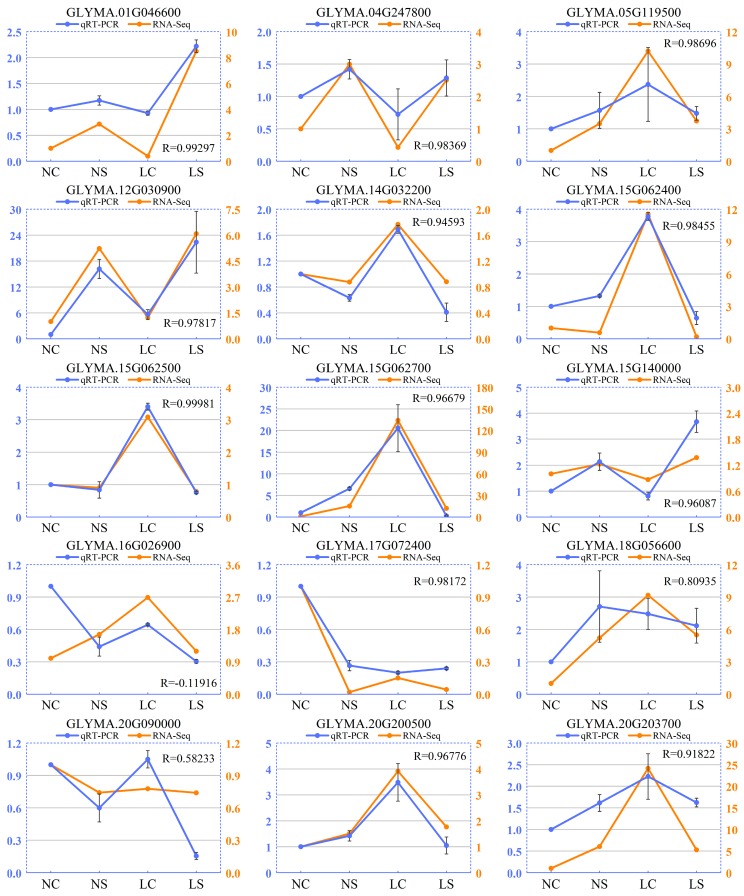
Comparison of the relative expression level change of 15 selected DEGs by qRT-PCR and RNA-seq. Left vertical axis coordinate is relative expression level of qRT-PCR (blue); right vertical axis coordinate is RPKM of RNA-Seq (orange). R-values are the correlation coefficients between qRT-PCR and RNA-seq. NS and NC refer to SMV infection and control, respectively, under normal light, while LS and LC refer to SMV infection and control, respectively, in the shade.

Here, we found that the expression of some genes was susceptible to light change, such as GLYMA_05G119500, GLYMA_14G032200, GLYMA_15G062700, and GLYMA_20G200500. The expression of those genes has changed dramatically in soybean in the shade (LC). However, infection with SMV has less effect on its gene expression. For other genes, their expression changed significantly after viral infection, but not sensitive to light change, such as GLYMA_01G046600 and GLYMA_15G140000. It suggested that there were differences between the effects of light and virus on soybean. Overall, qRT-PCR and RNA-Seq showed consistent expression patterns (Figure 5). The correlation coefficients between qRT-PCR and RNA-Seq were more than 0.9, except for GLYMA_16G026900 and GLYMA_20G090000. Minor expression differences might be due to differences in sensitivity between the two methods. These results indicated that the RNA-Seq data was reliable.

## 4. Conclusions

In this study, we simulated the shading environment by changing the light intensity and light quality. The response of soybean plants infected with SMV after 10 days under normal light and shade was studied. SMV infection severely limits the growth and development of soybeans, and the effects in the shade were more serious. At the transcriptional level, SMV infecting soybeans under normal light activates plant defense-related pathways, while expression of many genes was repressed in the shade, especially immune-related genes. Our study revealed that the defense response of soybean to SMV cannot be effectively activated in the shade and provided a basis for further study of the molecular mechanism of soybean and SMV interaction in the shade.

## Figures and Tables

**Table 1:** Overlapped DEGs related to plant-pathogen interactions under two light conditions.

Gene ID	Description	Log2FC (NS/NC)	Log2FC (LS/LC)
GLYMA_05G234600	MYB transcription factor MYB84	5.33	−3.24
GLYMA_18G056600	WRKY transcription factor 62	5.21	−3.66
GLYMA_09G038900	MYB transcription factor MYB13	5.06	1.74
GLYMA_06G187300	protein EDS1L	4.01	−2.16
GLYMA_20G034200	uncharacterized LOC100526868	3.94	−2.59
GLYMA_16G218300	probable cyclic nucleotide-gated ion channel 20	3.83	−1.29
GLYMA_02G270700	chitin elicitor receptor kinase 1-like	3.76	−1.16
GLYMA_19G214900	MYB transcription factor MYB111	3.20	2.40
GLYMA_09G210600	disease resistance protein RPM1	3.10	−1.92
GLYMA_06G034700	probable calcium-binding protein CML41	2.90	−2.53
GLYMA_20G209700	MYB/HD-like transcription factor	2.81	−3.06
GLYMA_10G180800	MYB29 protein	2.25	−3.85
GLYMA_18G208800	probable WRKY transcription factor 33	2.23	−3.83
GLYMA_03G042700	probable WRKY transcription factor 33	1.99	−2.70
GLYMA_14G222000	calcium-dependent protein kinase 29	1.97	1.79
GLYMA_05G119500	BRASSINOSTEROID INSENSITIVE 1-associated receptor kinase 1	1.80	−1.45
GLYMA_10G230000	protein SGT1 homolog B-like	1.73	−1.51
GLYMA_02G244600	MYB transcription factor MYB20	1.65	1.14
GLYMA_06G187400	protein EDS1-like	1.55	−1.01
GLYMA_19G255300	cyclic nucleotide-gated ion channel 1	1.35	−1.20
GLYMA_02G059600	putative calcium-binding protein	1.13	−1.11
GLYMA_14G156300	calcium-binding EF-hand family protein	−2.18	−1.76
GLYMA_16G178800	heat shock protein 90-A2	−5.23	−1.33
GLYMA_09G131500	heat shock protein 83	−7.64	−2.66

NS and NC refer to SMV infection and control, respectively, under normal light, while LS and LC refer to SMV infection and control, respectively, in the shade.

**Table 2:** DEGs involved in plant defense hormone signaling under two light conditions.

Gene ID	Description	Log2FC (NS/NC)	Log2FC (LS/LC)	Pathway
GLYMA_10G186800	ethylene-responsive transcription factor 1B	2.71	−1.21	ETH
GLYMA_04G147000	EIN3-binding F-box protein 1	2.70	-	
GLYMA_20G203700	ethylene-responsive transcription factor 1B	2.59	−2.19	
GLYMA_02G006200	ethylene-responsive transcription factor 1B	-	−6.71	
GLYMA_10G036700	ethylene-responsive transcription factor 1B	-	−2.88	
GLYMA_10G007000	ethylene-responsive transcription factor 1B	-	−2.68	
GLYMA_19G248900	ethylene-responsive transcription factor 1B	-	−1.55	
GLYMA_18G018400	putative ETHYLENE INSENSITIVE 3	1.67	2.24	
GLYMA_13G166200	EIN3-binding F-box protein 1	1.58	-	
GLYMA_20G202200	ethylene receptor 2	1.23	-	
GLYMA_13G076800	ETHYLENE INSENSITIVE 3-like 1 protein	1.15	-	
GLYMA_10G188500	ethylene receptor	1.07	-	
GLYMA_16G020500	transcription factor MYC2	2.49	-	JA
GLYMA_17G209000	transcription factor MYC2	-	−1.52	
GLYMA_13G112000	Jasmonate ZIM domain-containing protein	1.69	-	
GLYMA_16G026900	jasmonic acid-amido synthetase JAR1	-	−1.17	
GLYMA_18G030200	coronatine-insensitive protein 1	−1.31	-	
GLYMA_15G062300	pathogenesis-related protein 1-like protein	2.7	-	SA
GLYMA_15G062400	pathogenesis-related protein 1	-	−5.91	
GLYMA_15G062700	pathogenesis-related protein 1	-	−3.48	
GLYMA_15G062500	pathogenesis-related protein 1	-	−1.97	
GLYMA_09G020800	NPR1-1 protein	-	−1.05	
GLYMA_14G031300	regulatory protein NPR3	2.31	-	
GLYMA_02G283300	regulatory protein NPR3	1.04	-	
GLYMA_03G128600	regulatory protein NPR5	-	1.99	
GLYMA_05G182500	transcription factor TGA1-like	−1.06	-	
GLYMA_18G020900	transcription factor TGA4-like	-	−1.1	
GLYMA_14G167000	transcription factor TGA7	-	1.44	
GLYMA_13G085100	transcription factor bZIP83	-	1.08	

ETH, JA, and SA refer to ethylene, jasmonic acid, and salicylic acid. NS and NC refer to SMV infection and control, respectively, under normal light, while LS and LC refer to SMV infection and control, respectively, in the shade.

## Supplementary Materials

The following are available online at https://www.mdpi.com/1999-4915/11/9/793/s1, Figure S1. Plant pathogen interaction pathway map between NC vs NS. Figure S2. Plant pathogen interaction pathway map between LC vs LS. Figure S3. Plant defense hormone signal pathway map between NC vs NS. Figure S4. Plant defense hormone signal pathway map between LC vs LS. Table S1. Primers used for qRT-PCR validation. Table S2. Statistics of raw data. Table S3. Statistics of clean reads. Table S4. Statistics of RNA-Seq map. Table S5. Differentially expressed genes involved in plant-pathogen interaction (ko04626) pathway between NC and NS. Table S6. Differentially expressed genes involved in plant-pathogen interaction (ko04626) pathway between LC and LS.

## Abbreviations

CaM	calmodulin
ChiVMV	chilli veinal mottle virus
CK	control
CMV	cucumber mosaic virus
DEGs	differentially expressed genes
DPI	days post-inoculation
EDS1	enhanced disease susceptibility 1
ERF	ethylene-responsive transcription factor
ETH	ethylene
GO	Gene Ontology
JA	jasmonic acid
JAZ	jasmonate ZIM-domain
KEGG	Kyoto Encyclopedia of Genes and Genomes
log2FC	log2Fold Change
LC	Control in the shade
LS	SMV infection in the shade
NC	Control under normal light
NS	SMV infection under normal light
PAR	photosynthetically active radiation
phyB	phytochrome B
PR1	pathogenesis-related 1
qRT-PCR	quantitative real-time PCR
R:FR	red:far red-light ratio
RPKM	reads per kb per million reads
SA	salicylic acid
SAS	shade avoidance syndrome
SMV	soybean mosaic virus
TF	transcription factor
TuMV	turnip mosaic virus
